# Predictors of *Candida auris* Infection in Previously Colonized Patients: A Retrospective Cohort Study from a Large Tertiary Reference Center

**DOI:** 10.3390/jof12060449

**Published:** 2026-06-19

**Authors:** Nadide Ergün, Sevim Selen Karabulut, Melda Türken, Bengü Tatar, Süheyla Serin Senger

**Affiliations:** 1 Department of Infectious Diseases and Clinical Microbiology, Izmir City Hospital, Izmir 35540, Türkiye; shizlisoy@hotmail.com (S.S.K.); meldaulusoy@gmail.com (M.T.); b.gtatar@hotmail.com (B.T.); suheyla.serin@gmail.com (S.S.S.); 2 Izmir Faculty of Medicine, University of Health Sciences, Izmir 35540, Türkiye

**Keywords:** *Candida auris*, colonization, bloodstream infection, antifungal resistance, echinocandin, intensive care unit, Turkey

## Abstract

*Candida auris* is a multidrug-resistant fungal pathogen associated with high mortality in healthcare settings. Although colonization is recognized as the harbinger of invasive infection, predicting which patients will develop bloodstream infection (BSI) and when this transition will occur remains a clinical challenge. In this study, patients aged ≥18 years with *C. auris* colonization identified at İzmir City Hospital between January 2023 and June 2025 were retrospectively analyzed. Colonization was confirmed by matrix-assisted laser desorption/ionization time-of-flight mass spectrometry (MALDI-TOF MS). Of 71 colonized patients (median age 65 years; 69.0% male; 93.0% intensive care unit (ICU)-admitted), 31 (43.7%) developed bloodstream infection (BSI). In-hospital mortality was 62.0%, rising to 74.2% in the BSI group, though this difference did not reach statistical significance (*p* = 0.105). Competing risks analysis using the Aalen–Johansen method showed a cumulative BSI incidence of 38.2% (95% confidence interval (CI): 28–50%) by day 10 and 43.0% (95% CI: 32–54%) by day 30 following colonization detection. On multivariate logistic regression, diabetes mellitus was the sole variable independently associated with a lower risk of BSI development (adjusted odds ratio (OR): 0.19; 95% CI: 0.06–0.68; *p* = 0.010); this finding was directionally consistent but did not reach statistical significance in the multivariable Fine–Gray competing risks model (subdistribution hazard ratio (SHR): 0.334; 95% CI: 0.108–1.040; *p* = 0.057). All 40 tested isolates had high fluconazole minimum inhibitory concentration (MIC) values; micafungin susceptibility was 92.5%, while anidulafungin resistance was observed in 32.5% of isolates. Our findings demonstrate that nearly half of colonized patients developed BSI, with no identifiable safe window for intervention, underscoring the necessity of sustained infection control measures and susceptibility-guided antifungal therapy.

## 1. Introduction

The clinical significance of *Candida auris* in intensive care settings is now well established; identification and infection control responses are increasingly standardized. However, a critical question remains unanswered: which colonized patients will progress to invasive infection, and within what timeframe? Addressing this question has direct implications for clinical decision-making and infection control practice.

*Candida auris* was first isolated in 2009 from the external ear canal of a hospitalized patient in Japan and has since caused nosocomial outbreaks in more than forty countries across six continents [1]. Although recent publications have proposed reclassification as *Candidozyma auris*, the established *Candida auris* nomenclature has been retained throughout this study in keeping with prevailing literature usage [2]. In 2022, the World Health Organization designated this pathogen as a critical priority on its fungal priority pathogens list [3]. Widespread fluconazole resistance, the capacity to persist on inanimate surfaces for prolonged periods, and efficient transmission within healthcare settings distinguish *C. auris* from other Candida species and contribute to its global public health significance [4].

Colonization occupies a dual role in the clinical course of *C. auris* infection: it marks the entry point and sustains an ongoing risk. In intensive care settings, candidemia has been reported to develop in approximately 17% of colonized patients by day 30 and in 25% by day 60 [5]. These rates underscore the clinical importance of colonization surveillance. However, the central challenge remains unresolved: identifying which colonized patients are most likely to progress to invasive infection and determining the timeframe during which this risk is greatest.

Several studies have attempted to identify predictors of progression from colonization to bloodstream infection. In a multicenter intensive care unit (ICU) cohort, central venous catheterization, mechanical ventilation, and a higher burden of comorbidities were more common among patients who subsequently developed candidemia, although none remained independently associated with progression after multivariable adjustment [5]. In contrast, renal replacement therapy, prior antifungal exposure, and prolonged intensive care unit stay were identified as significant risk factors in a large Indian cohort [6]. More recently, immunosuppression and broad-spectrum antibiotic exposure were reported as risk factors for invasive infection development among colonized patients [7]. Taken together, these studies suggest that progression from colonization to infection is multifactorial; however, the reported predictors vary substantially across healthcare systems and geographic regions. Differences in patient populations, screening strategies, infection prevention practices, and local epidemiology likely contribute to these inconsistencies. Consequently, no universally accepted risk stratification model currently exists. Furthermore, the temporal dynamics of progression from colonization to bloodstream infection have received comparatively little attention, despite their potential importance for surveillance and targeted preventive interventions [8].

*Candida auris* bloodstream infection (BSI) carries a heavy clinical burden. Overall mortality has been reported at approximately 39%, with BSI-specific mortality reaching 45% [9]. Early initiation of appropriate antifungal therapy, catheter removal, and source control are among the factors associated with improved survival [10], making timely prediction of progression from colonization to infection all the more critical.

From an antifungal susceptibility perspective, *C. auris* presents a particularly challenging profile. The organism frequently exhibits intrinsic resistance to fluconazole and variable susceptibility to amphotericin B, while resistance to echinocandins, although currently less common, is increasingly reported and is largely mediated by mutations in the *FKS1* gene [11]. The interpretation of susceptibility testing has historically been complicated by the lack of species-specific clinical breakpoints. The recent release of the European Committee on Antimicrobial Susceptibility Testing (EUCAST) version 6.0 (2025), which introduced species-specific breakpoints for *C. auris*, represents an important step toward standardizing susceptibility assessment and therapeutic decision-making [12]. Nevertheless, contemporary local susceptibility data remain limited in many regions, emphasizing the need for ongoing surveillance.

In Turkey, *C. auris* has moved well beyond sporadic occurrence. The first documented fungemia case was reported in 2021 [13]. A national study conducted during the COVID-19 pandemic identified *C. auris* as responsible for 9% of candidemia episodes [14], and Turkish centers have contributed to international multicenter investigations [10]. Nevertheless, a comprehensive national cohort study examining the dynamics of colonization-to-BSI progression, the associated risk factors, and the antifungal susceptibility profile against current breakpoints has not yet been published [11].

Such data are urgently needed to inform local infection control policies, antifungal stewardship programs, and empirical treatment decisions in Turkish tertiary-care centers. The present study aimed to identify independent risk factors for BSI development in a *C. auris* colonization cohort at a tertiary-care center, to characterize the temporal course of the colonization-to-infection transition, and to evaluate the antifungal susceptibility profile against current EUCAST breakpoints (v6.0, 2025) [15].

## 2. Materials and Methods

This was a retrospective, observational cohort study conducted at İzmir City Hospital between January 2023 and June 2025. İzmir City Hospital is one of the largest tertiary-care centers in the region, with 12 intensive care units comprising exclusively single-occupancy rooms and a total capacity of approximately 270 ICU beds. The study protocol was approved by the local clinical research ethics committee (approval no: 2025/397).

Surveillance was initiated following identification of an index *C. auris* case through a clinical blood culture in an adult ICU. Given the high frequency of inter-ICU patient transfers within our institution, outbreak response screening was extended to all adult ICUs. Active surveillance was performed using composite axillary and inguinal swab specimens. During the initial screening phase, 270 ICU patients were screened. Each screening round consisted of three consecutive surveillance cultures obtained at 24 h intervals, and patients were considered negative only if all three cultures were negative. Patients with at least one positive surveillance culture were classified as colonized. Positive patients were initially cohorted in a designated ICU. Subsequently, whenever a new clinical *C. auris* isolate was identified in any ICU or ward, targeted rescreening was performed in all patients currently hospitalized in the corresponding unit; a total of 15 additional screening rounds were conducted during the study period. Overall, approximately 630 patient screening encounters were performed during the surveillance period, including the initial ICU-wide screening and subsequent targeted outbreak response screening rounds.

Following implementation of enhanced infection control measures, colonized patients were managed in single-occupancy ICU rooms with reinforced contact precautions, dedicated equipment, and separation of shared items. Previously identified colonized patients were not routinely rescreened because of the prolonged persistence of *C. auris* colonization. Only surveillance-confirmed colonized patients were included in the study cohort; patients identified solely through clinical specimens were excluded.

**Figure 1:**
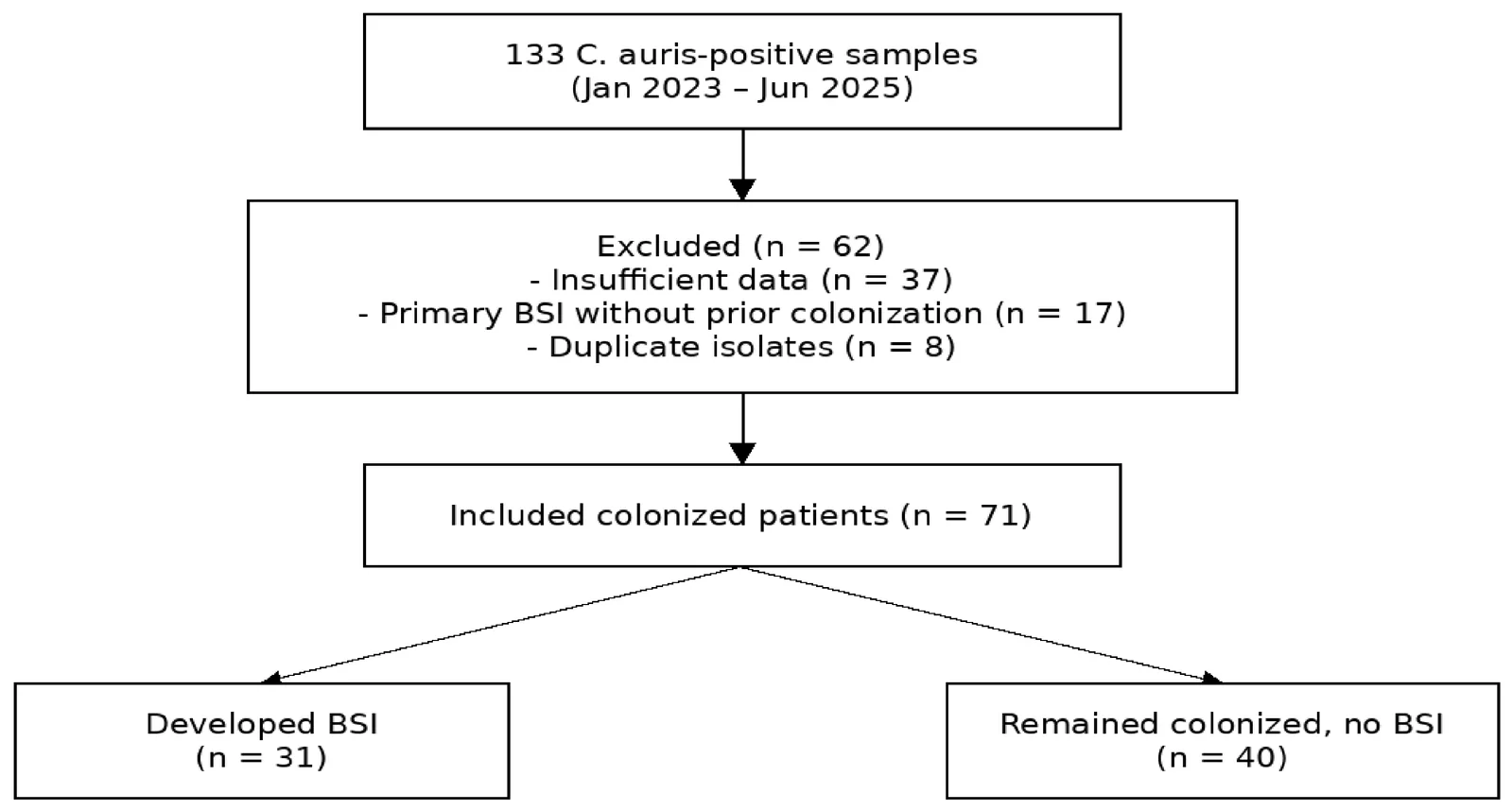
The flowchart of patient selection. A total of 133 *C. auris*-positive samples were identified between January 2023 and June 2025. Of these, 62 were excluded: 8 represented duplicate isolates from previously identified patients (only the first episode retained), 37 had insufficient clinical data, and 17 presented with primary bloodstream infection without prior documented colonization. The final cohort comprised 71 surveillance-confirmed colonized patients, of whom 31 subsequently developed BSI and 40 remained in the colonized, no-BSI group.

All patients aged 18 years or older with microbiologically confirmed *C. auris* colonization were included. Colonization was defined as isolation of *C. auris* from composite axillary and inguinal swab specimens confirmed by matrix-assisted laser desorption/ionization time-of-flight mass spectrometry (MALDI-TOF MS) (VITEK MS, bioMérieux, Marcy-l’Étoile, France). Bloodstream infection (BSI) was defined as the growth of *C. auris* in at least one blood culture set in the presence of compatible clinical signs. The patient selection flow diagram is presented in Figure 1.

Patients meeting any of the following criteria were excluded: (i) insufficient clinical follow-up data (*n* = 37), (ii) presentation with primary invasive infection in the absence of prior documented colonization (*n* = 17), and (iii) duplicate isolates from the same patient (*n* = 8), in which case only the first isolation episode was included.

All isolates were identified to species level by MALDI-TOF MS (VITEK MS, bioMérieux, Marcy-l’Étoile, France). Antifungal susceptibility testing was performed using the Sensititre YeastOne colorimetric broth microdilution system (Thermo Fisher Scientific, Cleveland, OH, USA). Susceptibility testing was performed on isolates obtained from clinical specimens (blood cultures in BSI patients, and urine or wound cultures in colonized patients with incidental clinical isolates); surveillance swab isolates were not routinely submitted for susceptibility testing in accordance with standard clinical practice. The test panel included fluconazole, voriconazole, itraconazole, posaconazole, amphotericin B, caspofungin, micafungin, and anidulafungin.

Minimum inhibitory concentration (MIC) values were interpreted according to EUCAST v6.0 (June 2025), which for the first time established species-specific clinical breakpoints for *C. auris* [15]. The epidemiological cut-off value (ECOFF) for amphotericin B is 2 mg/L; under EUCAST v6.0, the entire wild-type population falls within the susceptible, increased exposure (I) category (clinical breakpoints: S ≤ 0.001 mg/L, R > 2 mg/L), and all isolates with MICs at or below the ECOFF were considered clinically susceptible. It is recognized that the Sensititre YeastOne system systematically yields higher amphotericin B MIC values than reference methods, and this was taken into account during interpretation [12]. For echinocandins, a susceptibility breakpoint of S ≤ 0.25 mg/L and a resistance breakpoint of R > 0.25 mg/L were applied for both anidulafungin and micafungin. No species-specific EUCAST breakpoints are available for azoles or caspofungin in *C. auris*; the results for these agents are therefore presented descriptively. As the Sensititre YeastOne system is not the EUCAST reference method (E.Def 7.4), MIC-based categorical interpretations, particularly for echinocandins, were treated as exploratory.

Data were analyzed using SPSS (version 26.0; IBM Corp., Armonk, NY, USA). The normality of continuous variables was assessed with the Shapiro–Wilk test; data are presented as the median and interquartile range (IQR), and categorical variables as count and percentage. Between-group comparisons were performed using the Mann–Whitney U test, chi-square test, or Fisher’s exact test, as appropriate. Time from colonization detection to BSI development was analyzed using the Aalen–Johansen estimator, with death treated as a competing risk. To formally assess the effect of covariates on the subdistribution hazard of BSI, a multivariable Fine–Gray competing risks regression model was fitted, incorporating diabetes mellitus, malignancy, age, and ICU length of stay as covariates. Continuous variables were expressed per standard deviation to facilitate comparability. A Kaplan–Meier estimate was additionally generated as a sensitivity analysis. Competing risks analyses were performed using the cmprsk package (version 2.2-12) in R (version 4.6.0). Independent risk factors for BSI were identified by logistic regression analysis. Variables with *p* < 0.10 in univariate analysis (diabetes mellitus and ICU length of stay) and age, included on clinical grounds, were entered into the multivariate model. Statistical significance was defined as *p* < 0.05.

## 3. Results

A total of 71 patients met the diagnostic criteria for *C. auris* colonization during the study period. The median age was 65 years (IQR: 47–74), and 49 patients (69.0%) were male. The majority of patients (93.0%; *n* = 66) were admitted to the intensive care unit. Colonization was detected at a median of 37 days (IQR: 12–63) after hospital admission. In-hospital mortality was 62.0% (*n* = 44) (Table 1).

### 3.1. BSI Development and Clinical Comparison

Of 71 colonized patients, 31 (43.7%) developed *C. auris* bloodstream infection; 40 patients (56.3%) remained colonized without developing BSI. No statistically significant differences were observed between the two groups with respect to age, sex, ICU admission rate, or hospital length of stay. ICU length of stay was, however, significantly longer in the BSI group (median 62 days [IQR: 39–96] vs. 28 days [IQR: 13–78]; *p* = 0.023). Among comorbidities, the prevalence of diabetes mellitus was notably higher in patients who did not develop BSI (47.5% vs. 12.9%; *p* = 0.002). Malignancy appeared more frequent in the BSI group (12.9% vs. 2.5%), but this difference did not reach statistical significance (*p* = 0.160). Rates of central venous catheter use and mechanical ventilation did not differ significantly between groups (Table 2). The most common admission diagnoses were respiratory failure (*n* = 20, 28.2%), neurological disease (*n* = 19, 26.8%), and sepsis/septic shock (*n* = 13, 18.3%); remaining patients were admitted for trauma (*n* = 5, 7.0%), postoperative care (*n* = 4, 5.6%), cardiovascular disease (*n* = 3, 4.2%), or other reasons (*n* = 7, 9.9%). The distribution of admission diagnoses did not differ significantly between BSI and non-BSI groups (*p* = 0.290). Among the 31 patients who developed BSI, antifungal therapy was initiated in 29 (93.5%). The most frequently used agent was caspofungin (*n* = 19, 65.5%), followed by micafungin (*n* = 9, 31.0%) and anidulafungin (*n* = 1, 3.4%).

### 3.2. Risk Factor Analysis

On univariate logistic regression, the presence of diabetes mellitus was associated with a statistically significant reduction in BSI risk (odds ratio (OR): 0.16; 95% confidence interval (CI): 0.05–0.55; *p* = 0.004). No other variables, including age, sex, ICU length of stay, hospital length of stay, COPD, malignancy, central venous catheter, or mechanical ventilation, reached statistical significance (Table 3).

Diabetes mellitus was confirmed as independently associated with a lower risk of BSI development (adjusted OR: 0.19; 95% CI: 0.06–0.68; *p* = 0.010). Age and ICU length of stay lost statistical significance in the multivariate model (Table 4). In the competing risks analysis, diabetes mellitus showed a trend towards a lower risk of BSI development that did not reach statistical significance (subdistribution hazard ratio (SHR): 0.334; 95% CI: 0.108–1.040; *p* = 0.057), consistent in direction with the logistic regression findings (Table 5).

### 3.3. Time from Colonization to BSI

**Figure 2:**
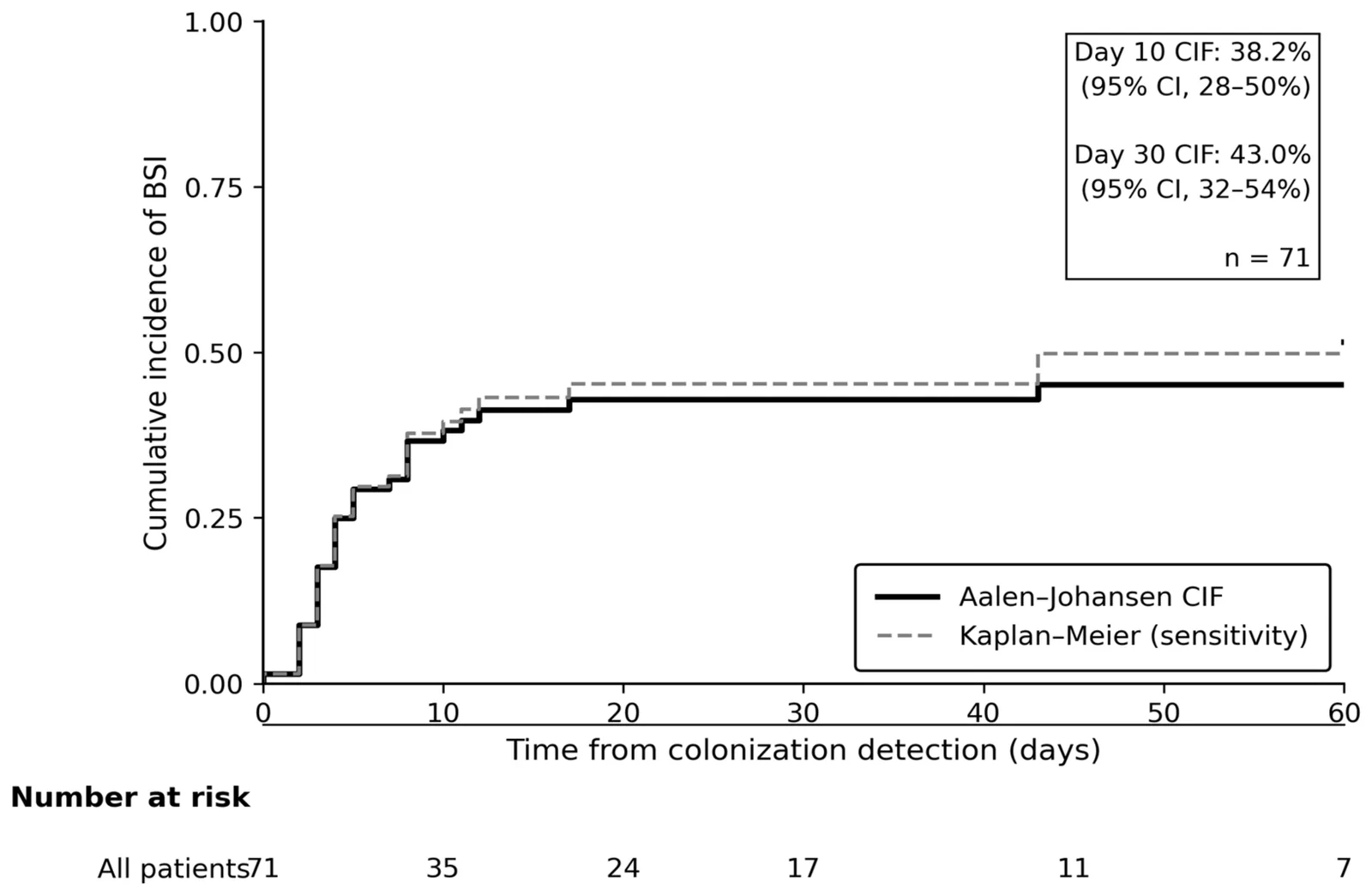
The cumulative incidence of bloodstream infection (BSI) among *Candida auris*–colonized patients (*n* = 71), estimated by the Aalen–Johansen method with death treated as a competing risk (solid line). A Kaplan–Meier estimate is shown as a sensitivity analysis (dashed line). Shaded areas represent 95% confidence intervals. Numbers at risk are shown below the x-axis. CIF, cumulative incidence function.

The cumulative incidence of BSI estimated using the Aalen–Johansen method with death as a competing risk reached 38.2% (95% CI: 28–50%) by day 10 and 43.0% (95% CI: 32–54%) by day 30 following colonization detection. The majority of BSI episodes occurred within the first 10 days, after which the cumulative incidence curve flattened substantially. A Kaplan–Meier sensitivity analysis yielded similar estimates, with a slight upward bias as expected when competing risks are not accounted for (Figure 2).

### 3.4. Antifungal Susceptibility Profile

Antifungal susceptibility testing by the Sensititre YeastOne colorimetric broth microdilution system was performed on 40 isolates. All isolates exhibited high fluconazole MIC values (median MIC: 128 µg/mL [IQR: 128.00–128.00]); as no EUCAST clinical breakpoint has been established for *C. auris* against azole agents, these results are presented descriptively.

Among echinocandins, micafungin susceptibility was high (92.5%; *n* = 37), whereas 13 isolates (32.5%) were classified as resistant to anidulafungin, yielding a susceptibility rate of 67.5% (*n* = 27). Given that the anidulafungin median MIC (0.25 µg/mL) falls exactly at the resistance breakpoint (R > 0.25 mg/L), and that the Sensititre YeastOne system is known to yield higher echinocandin MICs than the EUCAST reference method, this resistance rate likely reflects a methodological artifact rather than true *FKS*-mediated resistance. The biological plausibility of this interpretation is further supported by the concurrent high micafungin susceptibility rate (92.5%), given that *FKS*-mediated echinocandin resistance is expected to be class-wide.

**Figure 3:**
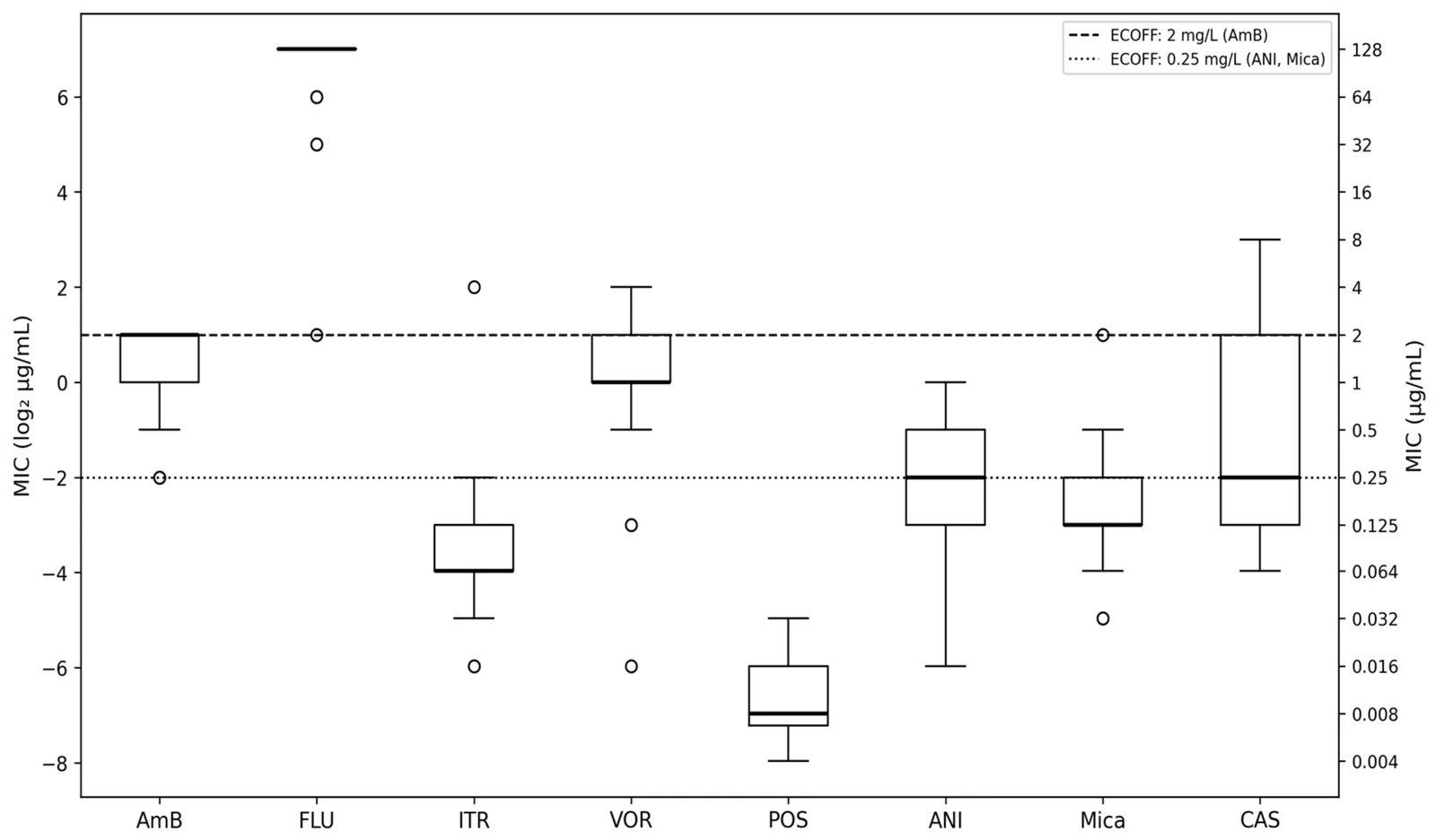
The distribution of log_2_-transformed minimum inhibitory concentration (MIC) values of *Candida auris* isolates (*n* = 40). Dashed lines indicate EUCAST v6.0 (2025) clinical breakpoints: the upper line represents the resistance breakpoint for amphotericin B (R > 2 mg/L; log_2_ = 1; all isolates below this threshold were classified as susceptible, increased exposure [I]); the lower line represents the susceptibility/resistance breakpoint for anidulafungin and micafungin (S ≤ 0.25 mg/L; R > 0.25 mg/L; log_2_ = −2). No EUCAST clinical breakpoints are currently available for fluconazole, itraconazole, voriconazole, posaconazole, or caspofungin in *C. auris*; MIC values for these agents are therefore presented descriptively. MIC testing was performed using the Sensititre YeastOne colorimetric broth microdilution system, a non-reference method; the results should be interpreted with caution.

All isolates (*n* = 40; 100%) had amphotericin B MIC values that did not exceed the resistance breakpoint (MIC range: 0.25–2.00 µg/mL). Per EUCAST v6.0, the entire wild-type population falls within the susceptible, increased exposure (I) category; results are therefore presented descriptively. No EUCAST breakpoint has been established for caspofungin in *C. auris*, and the results for this agent are likewise reported descriptively. MIC distributions are shown in Figure 3 (Table 6).

## 4. Discussion

We examined 71 patients in whom *C. auris* colonization was identified at a tertiary-care hospital; the majority (93.0%) were admitted to the intensive care unit. Nearly half of the colonized patients developed bloodstream infection, diabetes mellitus showed an unexpected inverse association with BSI risk, and resistance to anidulafungin was observed at a level that cannot be disregarded.

### 4.1. Colonization to BSI Progression

A BSI rate of 43.7% among colonized patients exceeds figures reported in the literature. Briano et al. reported rates of 17% by day 30 and 25% by day 60 in ICU patients [5]. The higher rate observed in our cohort is consistent with the clinical profile of our study population: 93% of patients were admitted to the ICU, rates of invasive procedures were high, and screening was initiated in response to an outbreak, meaning the cohort likely reflects a particularly severely ill patient group.

The Aalen–Johansen analysis demonstrated that BSI development occurred predominantly within the first 10 days following colonization detection, with cumulative incidence reaching 38.2% by day 10. The clinical message is unambiguous: there is no safe window for a colonized patient. The fact that infection emerged in most cases within the first 10 days indicates that clinical vigilance and infection control measures must be fully implemented from the moment colonization is detected [5,16]. These findings are broadly consistent with data from other colonization cohorts. Higher comorbidity burden, central venous catheter use, and mechanical ventilation have been reported as more prevalent among patients progressing to invasive infection, although these variables did not independently predict progression on multivariate analysis in several studies—a finding that mirrors our own results [14]. Prior antifungal and antibiotic exposure has also been repeatedly identified as a potential facilitator of progression; in the present cohort, prior antifungal exposure was evaluated but did not reach the threshold for inclusion in the multivariate model (*p* = 0.263), precluding formal assessment of its independent contribution.

### 4.2. Diabetes Mellitus and BSI Risk: A Non-Significant Association in Competing Risks Analysis

We observed an apparent inverse association between diabetes mellitus and BSI development on multivariate logistic regression (adjusted OR: 0.19; *p* = 0.010); however, this association was attenuated and did not reach statistical significance in the subdistribution hazard model (SHR: 0.334; 95% CI: 0.108–1.040; *p* = 0.057), which is generally considered the preferred approach in the presence of competing risks. Accordingly, this finding should be interpreted with caution and should not be construed as evidence of a true biological protective effect of diabetes mellitus against *C. auris* BSI.

The attenuation observed in the Fine–Gray model suggests that the apparent association observed in logistic regression may be influenced by the competing risk of death among non-diabetic patients, who may have died before BSI could develop. Diabetes mellitus is generally regarded as a risk factor for invasive fungal infections; therefore, a true protective biological effect appears unlikely. One possible explanation is that diabetic patients may have received closer clinical monitoring and earlier interventions. However, we found no significant difference in prior antifungal exposure between groups (*p* = 0.263), which does not support this hypothesis. Residual confounding cannot be excluded, and prospective studies are needed to clarify whether any true relationship exists.

### 4.3. Echinocandin Resistance

Echinocandins are the first-line treatment for *C. auris* infections [10,11]. Micafungin susceptibility was high at 92.5%, whereas anidulafungin resistance was detected in 32.5% of isolates, a rate that warrants attention. Echinocandin resistance is generally reported at 0–8% in the literature [4,8]; the rate observed in our cohort substantially exceeds this range. This discrepancy cannot be fully explained, as genotypic analysis was not performed within the scope of this study. While *FKS1* gene mutations represent the principal known resistance mechanism [11], molecular confirmation is required. The marked difference in susceptibility between micafungin and anidulafungin, two agents within the same class, underlines the indispensability of individual susceptibility-guided therapy even when selecting within a single drug class.

### 4.4. Mortality

In-hospital mortality was 74.2% in the BSI group and 52.5% among colonized patients without BSI; this difference did not reach statistical significance (*p* = 0.105). The high mortality observed in both groups is consistent with the clinical characteristics of the cohort. These were critically ill patients with serious underlying comorbidities. Isolating mortality directly attributable to *C. auris* in such a high-risk population is inherently difficult, a challenge consistently acknowledged in the literature [8,9].

### 4.5. Turkey Context

*Candida auris* has become increasingly established in Turkey, with growing evidence of widespread healthcare-associated transmission, though comprehensive national surveillance data are still accumulating. Mandatory reporting has been in effect since 10 September 2024, and national surveillance data continue to accumulate [11]. The present study is among the first national cohort studies from Turkey to comprehensively examine the dynamics of colonization-to-BSI progression and the antifungal susceptibility profile against current EUCAST breakpoints. The comparability of our findings with the national surveillance system lends them relevance to the broader epidemiological picture [11].

### 4.6. Limitations

This study has several limitations. The retrospective design precludes the establishment of causal relationships. The single-center setting limits the generalizability of the findings. Furthermore, as active screening was initiated in response to an identified outbreak, the cohort may disproportionately represent severely ill patients, and the observed BSI rate may not reflect the true background rate of progression in all colonized patients. An additional consideration is the relatively long interval between ICU admission and detection of *C. auris* colonization. This finding reflects the outbreak response nature of the surveillance program rather than delayed routine screening. Active surveillance was initiated following identification of an index case, and many patients had already been hospitalized before the first screening rounds were implemented. Therefore, the observed interval should not be interpreted as the natural time required for acquisition or detection of colonization.

The use of culture and MALDI-TOF MS rather than polymerase chain reaction (PCR), the EUCAST reference method for colonization screening, may have resulted in some colonized cases being missed. The use of the Sensititre YeastOne system, which is not the EUCAST reference microdilution method, may have produced MIC values that do not fully align with reference results. The absence of molecular typing and genotypic resistance analysis limits the mechanistic interpretation of the resistance patterns observed. To address the competing risk of death, cumulative BSI incidence was estimated using the Aalen–Johansen method and a multivariable Fine–Gray subdistribution hazard model was fitted; the Kaplan–Meier estimate was retained as a sensitivity analysis. Prior antifungal exposure was evaluated in the univariate analysis but did not meet the threshold for inclusion in the multivariate model (*p* = 0.263); accordingly, its independent contribution to BSI risk could not be formally assessed. Data on time to antifungal initiation and treatment duration were likewise unavailable and could not be assessed. Compared with included patients, excluded patients were significantly older (median age 75 vs. 65 years; *p* = 0.019), though sex distribution and ICU admission rates were similar (*p* = 0.522 and *p* = 0.506, respectively). Exclusion was attributable to insufficient clinical follow-up data rather than clinical characteristics, and is unlikely to have introduced systematic bias into the primary analysis.

## 5. Conclusions

*Candida auris* colonization represents a persistent and underestimated threat in critically ill patients. This study demonstrates that nearly half of colonized patients progressed to bloodstream infection, and that this transition occurred predominantly within the first ten days following colonization detection, leaving no identifiable safe window for intervention. Competing risks analysis using the Aalen–Johansen estimator confirmed that infection control measures and clinical vigilance must be maintained without interruption from the moment colonization is identified.

The inverse association between diabetes mellitus and BSI development most likely reflects the higher competing risk of death among non-diabetic patients, who may have died before BSI could develop, rather than a true biological protective effect. This interpretation is supported by the attenuation of the association in the Fine–Gray competing risks model and should not be construed as evidence of diabetic protection against invasive candidiasis.

Antifungal susceptibility findings underscore that a uniform empirical approach to *C. auris* is insufficient. While micafungin susceptibility was reassuringly high, the substantial anidulafungin resistance rate observed likely amplified by methodological factors inherent to the Sensititre YeastOne system highlights that individual susceptibility-guided therapy is not optional but indispensable, even when selecting within a single drug class.

As *C. auris* becomes increasingly entrenched in Turkish healthcare settings, strengthening national surveillance, expanding active screening programs, and maintaining continuous monitoring of antifungal susceptibility profiles must be regarded as urgent clinical and public health priorities.

## Figures and Tables

**Table 1:** Baseline characteristics of patients colonized with *Candida auris* (*n* = 71).

Variable	Total (*n* = 71)
Age, years, median (IQR)	65 (47–74)
Male sex, *n* (%)	49 (69.0)
ICU admission, *n* (%)	66 (93.0)
Days from hospital admission to colonization, median (IQR)	37 (12–63)
Overall mortality, *n* (%)	44 (62.0)

Abbreviations: IQR, interquartile range; ICU, intensive care unit.

**Table 2:** Comparison of clinical characteristics between *Candida auris* bloodstream infection (BSI) and colonization groups.

Variable	No BSI (*n* = 40)	BSI (*n* = 31)	*p*-Value
**Demographics**			
Age, years, median (IQR)	69 (45–78)	60 (47–69)	0.107
Male sex, *n* (%)	28 (70.0)	21 (67.7)	0.840
**Clinical characteristics**			
ICU admission, *n* (%)	36 (90.0)	30 (96.8)	0.270
Days from admission to colonization, median (IQR)	31 (4–70)	37 (22–57)	0.360
ICU length of stay, days, median (IQR)	28 (13–78)	62 (39–96)	0.023
Hospital length of stay, days, median (IQR)	59 (29–106)	79 (57–115)	0.198
Mortality, *n* (%)	21 (52.5)	23 (74.2)	0.105
**Comorbidities**			
Diabetes mellitus, *n* (%)	19 (47.5)	4 (12.9)	0.002
COPD, *n* (%)	5 (12.5)	5 (16.1)	0.660
Malignancy, *n* (%)	1 (2.5)	4 (12.9)	0.160
**Treatment history**			
Steroid use, *n* (%)	7 (17.5)	8 (25.8)	0.559
Non-steroid immunosuppressive therapy, *n* (%)	5 (12.5)	2 (6.5)	0.457
Prior antifungal exposure, *n* (%)	12 (30.0)	5 (16.1)	0.263
**Invasive procedures**			
Central venous catheter, *n* (%)	35 (87.5)	29 (93.5)	0.222
Mechanical ventilation, *n* (%)	30 (75.0)	22 (71.0)	0.182

Abbreviations: BSI, bloodstream infection; IQR, interquartile range; ICU, intensive care unit; COPD, chronic obstructive pulmonary disease. Continuous variables were compared using the Mann–Whitney U test; categorical variables were compared using the χ^2^ test or Fisher’s exact test, as appropriate.

**Table 3:** Univariate logistic regression analysis of risk factors for bloodstream infection (BSI) among patients colonized with *Candida auris*.

Variable	OR (95% CI)	*p*-Value
Age, per year	0.99 (0.96–1.01)	0.190
Male sex	0.90 (0.33–2.48)	0.838
ICU length of stay, days	1.01 (1.00–1.02)	0.080
Hospital length of stay, days	1.00 (1.00–1.01)	0.412
Diabetes mellitus	0.16 (0.05–0.55)	0.004
COPD	1.35 (0.35–5.14)	0.664
Malignancy	5.78 (0.61–54.58)	0.126
Central venous catheter	4.29 (0.47–38.75)	0.195
Mechanical ventilation	2.21 (0.82–5.97)	0.117

Abbreviations: OR, odds ratio; CI, confidence interval; BSI, bloodstream infection; ICU, intensive care unit; COPD, chronic obstructive pulmonary disease. Outcome: development of BSI among colonized patients.

**Table 4:** Multivariate logistic regression analysis of independent risk factors for bloodstream infection (BSI) among patients colonized with *Candida auris*.

Variable	Adjusted OR (95% CI)	*p*-Value
Age, per year	1.00 (0.97–1.02)	0.790
ICU length of stay, days	1.00 (1.00–1.01)	0.317
Diabetes mellitus	0.19 (0.06–0.68)	0.010

Abbreviations: OR, odds ratio; CI, confidence interval; BSI, bloodstream infection; ICU, intensive care unit. Variables with *p* < 0.10 in univariate analysis were included in the multivariate model. Age was additionally included as a clinically relevant covariate regardless of univariate *p*-value. Outcome: development of BSI among colonized patients.

**Table 5:** Multivariable Fine–Gray Subdistribution Hazard Model for Progression to BSI among *C. auris*-Colonized Patients (*n* = 71).

Variable	SHR	95% CI	*p*-Value
Diabetes mellitus	0.334	0.108–1.040	0.057
Malignancy	1.907	0.741–4.910	0.180
Age (per SD)	0.868	0.617–1.220	0.420
ICU LOS (per SD)	1.068	0.802–1.420	0.650

Abbreviations: SHR, subdistribution hazard ratio; CI, confidence interval; ICU, intensive care unit; LOS, length of stay; SD, standard deviation; BSI, bloodstream infection. Competing risks analysis was performed using the Fine–Gray subdistribution hazard model implemented in the cmprsk package (version 2.2-12) in R (version 4.6.0). Age and ICU length of stay are expressed per standard deviation. A *p*-value <0.05 was considered statistically significant.

**Table 6:** Antifungal susceptibility profiles of *Candida auris* isolates (*n* = 40) interpreted according to EUCAST Antifungal Breakpoints and ECOFF overview (v6.0), 2025.

Antifungal Agent	MIC Range (µg/mL)	Median MIC (IQR) µg/mL	Interpretation
Amphotericin B ^†^	0.25–2.00	2.00 (1.00–2.00)	Descriptive (I category) ^‡^
Fluconazole ^§^	2.00–128.00	128.00 (128.00–128.00)	Descriptive (IE)
Itraconazole ^§^	0.016–4.00	0.064 (0.064–0.125)	Descriptive (IE)
Voriconazole ^§^	0.016–4.00	1.00 (1.00–2.00)	Descriptive (IE)
Posaconazole ^§^	0.004–0.032	0.008 (0.004–0.016)	Descriptive (IE)
Anidulafungin ^¶^	0.016–1.00	0.25 (0.125–0.50)	S: 27 (67.5%); R: 13 (32.5%)
Micafungin ^¶^	0.032–2.00	0.125 (0.125–0.25)	S: 37 (92.5%); R: 3 (7.5%)
Caspofungin ^a^	0.064–8.00	0.25 (0.125–1.00)	Descriptive

Abbreviations: IQR, interquartile range; MIC, minimum inhibitory concentration; S, susceptible (standard dosing regimen); R, resistant; I, susceptible, increased exposure; IE, insufficient evidence; ND, not determined. MIC testing was performed using the Sensititre YeastOne colorimetric broth microdilution system (Thermo Fisher Scientific, Cleveland, OH, USA). The results were interpreted according to EUCAST Antifungal Breakpoints and ECOFF overview version 6.0 (June 2025). As this system is a non-reference method, MIC-based categorical interpretations should be made cautiously. ^†^ Amphotericin B: EUCAST has established a clinical S breakpoint of ≤0.001 mg/L and R breakpoint of >2 mg/L for *C. auris*, with ECOFF = 2 mg/L. All 40 isolates (100%) had MIC values ≤ 2 mg/L; none exceeded the resistance breakpoint. The entire wild-type population falls within the susceptible, increased exposure (I) category. The results are therefore presented descriptively. ^‡^ I (susceptible, increased exposure): high likelihood of therapeutic success when drug exposure is increased by adjusting the dosing regimen (recommended: liposomal amphotericin B 5 mg/kg/day). ^§^ Azoles: EUCAST lists insufficient evidence (IE) for fluconazole against *C. auris*; no ECOFF or clinical breakpoint is defined. For itraconazole, voriconazole, and posaconazole, breakpoints are listed as not determined (ND) for *C. auris*. Accordingly, MIC values are reported descriptively for all azole agents. ^¶^ Anidulafungin and micafungin: EUCAST defines both the ECOFF (WT ≤0.25 mg/L) and the clinical breakpoints (S ≤0.25 mg/L; R >0.25 mg/L) at the same value for *C. auris*. Isolates with MIC ≤0.25 mg/L are categorized as susceptible; those with MIC >0.25 mg/L as resistant. The anidulafungin resistance rate (32.5%) should be interpreted with caution: the median MIC falls exactly at the breakpoint boundary, and the Sensititre YeastOne system is known to yield higher echinocandin MICs than the EUCAST reference method. This finding likely represents a methodological artifact; the concurrent micafungin susceptibility rate (92.5%) supports this interpretation, as *FKS*-mediated echinocandin resistance is expected to be class-wide. ^a^ Caspofungin: No EUCAST breakpoints or ECOFF established for *C. auris* due to significant inter-laboratory variability in MIC determination. Per EUCAST guidance, isolates susceptible to both anidulafungin and micafungin may be considered susceptible to caspofungin. Results are presented descriptively.

## Data Availability

The data presented in this study are available upon reasonable request from the corresponding author. The data are not publicly available due to privacy restrictions in accordance with applicable personal data protection legislation.

## Abbreviations

The following abbreviations are used in this manuscript:

AmB	Amphotericin B
ANI	Anidulafungin
BSI	Bloodstream infection
CAS	Caspofungin
CI	Confidence interval
COPD	Chronic obstructive pulmonary disease
ECOFF	Epidemiological cut-off value
EUCAST	European Committee on Antimicrobial Susceptibility Testing
FLU	Fluconazole
ICU	Intensive care unit
IQR	Interquartile range
ITR	Itraconazole
MALDI-TOF MS	Matrix-assisted laser desorption/ionization time-of-flight mass spectrometry
Mica	Micafungin
MIC	Minimum inhibitory concentration
OR	Odds ratio
PCR	Polymerase chain reaction
POS	Posaconazole
VOR	Voriconazole
WHO	World Health Organization
